# Retinal vascular occlusion risks during the COVID-19 pandemic and after SARS-CoV-2 infection

**DOI:** 10.1038/s41598-023-44199-z

**Published:** 2023-10-06

**Authors:** Hyo Song Park, Sunyeup Kim, Christopher Seungkyu Lee, Suk Ho Byeon, Sung Soo Kim, Seung Won Lee, Yong Joon Kim

**Affiliations:** 1 Department of Ophthalmology, College of Medicine, Soonchunhyang University, Cheonan, South Korea; 2 Department of Ophthalmology, Soonchunhyang University Hospital Bucheon, Bucheon, South Korea; 3 Department of Medical AI, Sungkyunkwan University School of Medicine, Suwon, South Korea; 4 Department of Ophthalmology, The Institute of Vision Research, Yonsei University College of Medicine, Seoul, South Korea; 5 Department of Precision Medicine, Sungkyunkwan University School of Medicine, Suwon, South Korea; 6 Department of Ophthalmology, Institute of Vision Research, Severance Hospital, Yonsei University College of Medicine, Seoul, South Korea

**Keywords:** Diseases, Medical research, Risk factors

## Abstract

The coronavirus disease 2019 (COVID-19) has been reported to affect vascular networks including the eye. However, evidence on the causal relationship between COVID-19 infection and retinal vascular occlusions remains limited. This study aimed to determine the change in retinal vascular occlusion incidence during COVID-19 era and whether severe acute respiratory syndrome coronavirus 2 (SARS-CoV-2) infection induces retinal vascular occlusion. Retinal vein occlusion (RVO) and retinal artery occlusion (RAO) incidences during 2018–2019 and 2020–July 2021 were compared, those in confirmed and suspected COVID-19 patients diagnosed from 2020 to January 2021 were calculated, and those in COVID-19 patients during 180 days prior and 180 days after diagnosis were assessed. Additionally, the standardized incidence ratio of RVOs in COVID-19 patients was analyzed. Incidence rates per 100,000 people/year of RVO during 2018–2019 and 2020–2021 was 102.0 and 98.8, respectively. RAO incidence rates during 2018–2019 and 2020–2021 were 11.7 and 12.0, respectively. In both confirmed and suspected COVID-19 patients, the incidence of RVO and RAO did not change significantly from 180 days before to after diagnosis in the adjusted model. RVO incidence slightly decreased while RAO incidence increased during the COVID-19 pandemic. SARS-CoV-2 infection did not significantly increase RVO or RAO incidence.

## Introduction

With the advent of the coronavirus disease 2019 (COVID-19) pandemic, increasing interest and concern have been cast on the natural history, including complications, of the disease and its possible correlation with other diseases. Therefore, as COVID-19 was related to increased incidence of major arterial or venous thromboembolic events, its effect on other vascular networks, including the eye, has been of interest^1–3^.

Few studies have reported the effect of COVID-19 on retinal vasculature with imaging modalities such as optical coherence tomography or optical coherence tomography angiography. They have suggested the possibility of increased risk of retinal vascular complications resulting from decreased vascular density^4–6^. Furthermore, multiple case reports and series have reported the occurrence of retinal vascular occlusive events, including retinal vascular occlusions in various shapes and forms as a result of COVID-19 infection^7–15^.

However, there has been limited evidence on the causal relationship between COVID-19 infection and retinal vascular occlusions and the increase in the number of retinal vascular occlusion cases triggered by COVID-19. Furthermore, the COVID-19 pandemic induced major changes in global lifestyles, such as wearing masks, which could have influenced the change in the dynamics of retinal vascular occlusive disease. Therefore, a nationwide cohort study was conducted to analyze this change in the incidence rate of retinal vascular complications caused by COVID-19 infection using the Korean national health insurance claims-based database and Korean national COVID-19 registers. In addition, this study aimed to determine whether the incidence of retinal vascular occlusion increased during the COVID-19 pandemic era and whether severe acute respiratory syndrome coronavirus 2 (SARS-CoV-2) infection caused retinal vascular occlusion.

## Methods

The study adhered to the Declaration of Helsinki and received approval from the institutional review board of Severance Hospital (IRB No. 4-2021-0804). The data used for this study were approved by the Korean National Health Insurance Review & Assessment Service (M20220222838). Owing to the nature of the data used in the study, explained in detail in the following section, the need for informed consent was waived by the institutional review board of Severance Hospital (IRB No. 4-2021-0804).

### Data source

This study was based on a Korean national health insurance claims-based database and national COVID-19 registers. The data were obtained through the Healthcare Bigdata Hub of the Health Insurance Review & Assessment Service of Korea (https://opendata.hira.or.kr/), Korea Centers for Disease Control and Prevention, and Ministry of Health and Welfare. During the COVID-19 pandemic, the Korean government provided obligatory and complementary healthcare and insurance to every COVID-19 patient. The national health insurance claims-based database consisted of demographics and data from inpatient and outpatient healthcare visits, including data on healthcare and pharmaceutical visits, prescriptions, diagnoses, and procedures. The Korean government anonymized all patient data to ensure patient confidentiality. This large nationwide cohort included all patients who were diagnosed with retinal vascular occlusions as per the International Standard Classification of Disease (ICD)-10 with codes of H34.1 (central retinal artery occlusion [RAO]), H34.2 (branch RAO), or H34.8 (retinal vein occlusion [RVO]) from January 2016 to July 2021 and patients who had undergone SARS-CoV-2 testing in South Korea from January 2020 to January 2021. The first confirmed case of COVID-19 was reported in South Korea in January 2020, and the monthly confirmed cases of COVID-19 from January 2020 to January 2021 are presented in Supplementary Fig. 1.

### Study population

The study was based on three cohorts. Cohort A included retinal vascular occlusion patients with the diagnosis of codes H34.1, H34.2, or H34.8 from January 2016 to July 2021. Cohorts B and C included patients who were diagnosed with U07.1 (COVID-19 confirmed, virus identified) or U07.2 (COVID-19 suspected, virus not identified) until January 2021 and were followed up for 180 days after the diagnosis to evaluate RAO or RVO occurrence. Cohort B comprised patients who were never diagnosed with retinal vascular occlusion with ICD-10 codes of H34.1, H34.2, or H34.8 180 days before the diagnosis of U07.1 or U07.2. Cohort C included patients who were never diagnosed with retinal vascular occlusion with codes H34.1, H34.2, or H34.8 before the diagnosis of U07.1 or U07.2. SARS-CoV-2 tests based on reverse transcription-polymerase chain reaction assays of nasal or pharyngeal swabs authorized by the Korea Centers for Disease Control and Prevention established by the World Health Organization guidelines were required for the diagnosis of U07.1. In Cohorts B and C, patients with the diagnoses of both U07.1 and U07.2 were included in the U07.1 group and were excluded from the U07.2 group.

In a sub-analysis that assessed the severity of COVID-19, severe COVID-19 cases were defined as patients who were admitted to intensive care unit or needed oxygen therapy, including conventional oxygen therapy, high-flow nasal cannula oxygen therapy, mechanical ventilation, or extracorporeal membrane oxygenation, with claim codes AJx, M0040, M0046, M5850, M5857, M5858, M5860, or O1901–O1904. In another sub-analysis that assessed patients with coagulopathies, inherited or acquired hypercoagulable states were defined as patients who had received one of the following ICD-10 codes before the diagnosis of U07.1 or U07.2: secondary polycythemia (D75.1); polycythemia vera (D45); multiple myeloma (C90.0) Waldenstrom macroglobulinemia (C88.0); hyperhomocysteinemia (E72.1); lupus (D68.6); dysfibrinogemia (D68.2); primary thrombophilia (D68.5); or factor XII deficiency (D68.2).

### Exposure and outcomes

To assess whether there was a change in the incidence of retinal vascular occlusions during the COVID-19 pandemic, Cohort A was further divided according to time periods; 2016–2017 was used as a washout period to clarify new diagnoses during the following time periods, 2018–2019 was entitled as the “pre-COVID” era, and 2020 to July 2021 was considered as the “post-COVID” era. The incidence of RVOs and RAOs during pre-COVID and post-COVID periods was calculated and compared.

For the patients from Cohort B, which included patients who were never diagnosed with any of the ICD-10 codes of H34.1, H34.2, or H34.8 180 days before the diagnosis of U07.1 or U07.2, the incidence of new retinal vascular occlusions (RAO with H34.1 and H34.2 and RVO with H34.8) was compared between the pre-COVID-19 period of 180 days before diagnosis to the time point of diagnosis and post-infection period of until 180 days following the diagnosis.

For the patients in Cohort C, who were never diagnosed with H34.1, H34.2, or H34.8 before the diagnosis of U07.1 or U07.2, age and sex-adjusted standardized incidence ratio (SIR) of RAO (H34.1 and H34.2) and RVO (H34.8) were analyzed. For the calculation of SIR of retinal vascular occlusions in the population with the diagnosis of COVID-19, incidences of retinal vascular occlusions from 2018 to 2019 and from 2020 to 2021 were used as the reference. In addition, for the incidence rate and SIR derivation, the Korean population in the middle of the year, as calculated by Statistics Korea for that specific year, was applied.

### Covariates

Information on age; sex; comorbidities including diabetes mellitus (DM), hypertension, dyslipidemia, chronic kidney disease (CKD), and stroke; and region of residence (urban or rural) was gathered from insurance eligibility data. The presence of comorbidities was analyzed using one or more diagnoses with ICD-10 codes (Hypertension; I10, I11, I12, I13, I15, DM; E10, E11, E12, E13, E14, dyslipidemia; E78, CKD; N18, N19, stroke; I60, I61, I62, I63, I64).

### Statistics

In Cohort A, the incidence of RVOs and RAOs in 2018–2019 and 2020–July 2021 was calculated using the population estimated at the middle of the year and was compared using an independent t-test. In Cohort B, crude rate, adjusted incidence rate ratios (IRRs), and unadjusted IRRs of RVOs and RAOs 180 days before and after the diagnoses of U07.1 and U07.2 were calculated. For adjusted IRRs, multivariate analysis using covariates including age, sex, comorbidities (DM, hypertension, dyslipidemia, CKD, stroke), and residence (urban or rural) was conducted. For Cohort C, age-sex adjusted SIR of RVOs and RAOs after the diagnoses of U07.1 and U07.2 was derived. A p-value of < 0.05 was considered statistically significant.

Sub-analysis considering the severity of COVID-19 was conducted. For severe COVID-19 patients from Cohorts B and C, adjusted and unadjusted IRRs of RVOs and RAOs 180 days before and after the diagnoses of U07.1 and U07.2 and age-sex adjusted SIR of RVOs and RAOs after the diagnoses of U07.1 and U07.2 were calculated.

Another sub-analysis that assessed coagulopathy, which is a known risk factor for retinal vascular occlusion, was performed. For patients with inherited or acquired hypercoagulable states from Cohorts B and C, adjusted and unadjusted IRRs of RVOs and RAOs 180 days before and after the diagnoses of U07.1 and U07.2 and age-sex adjusted SIR of RVOs and RAOs after the diagnoses of U07.1 and U07.2 were calculated.

All statistical analyses were performed using SAS version 9.4 (SAS Institute Inc., Cary, NC, USA) and R version 3.5.3 (R Foundation for Statistical Computing, Vienna, Austria).

## Results

### Incidence of retinal vascular occlusion before and after the COVID-19 outbreak

**Figure 1:**
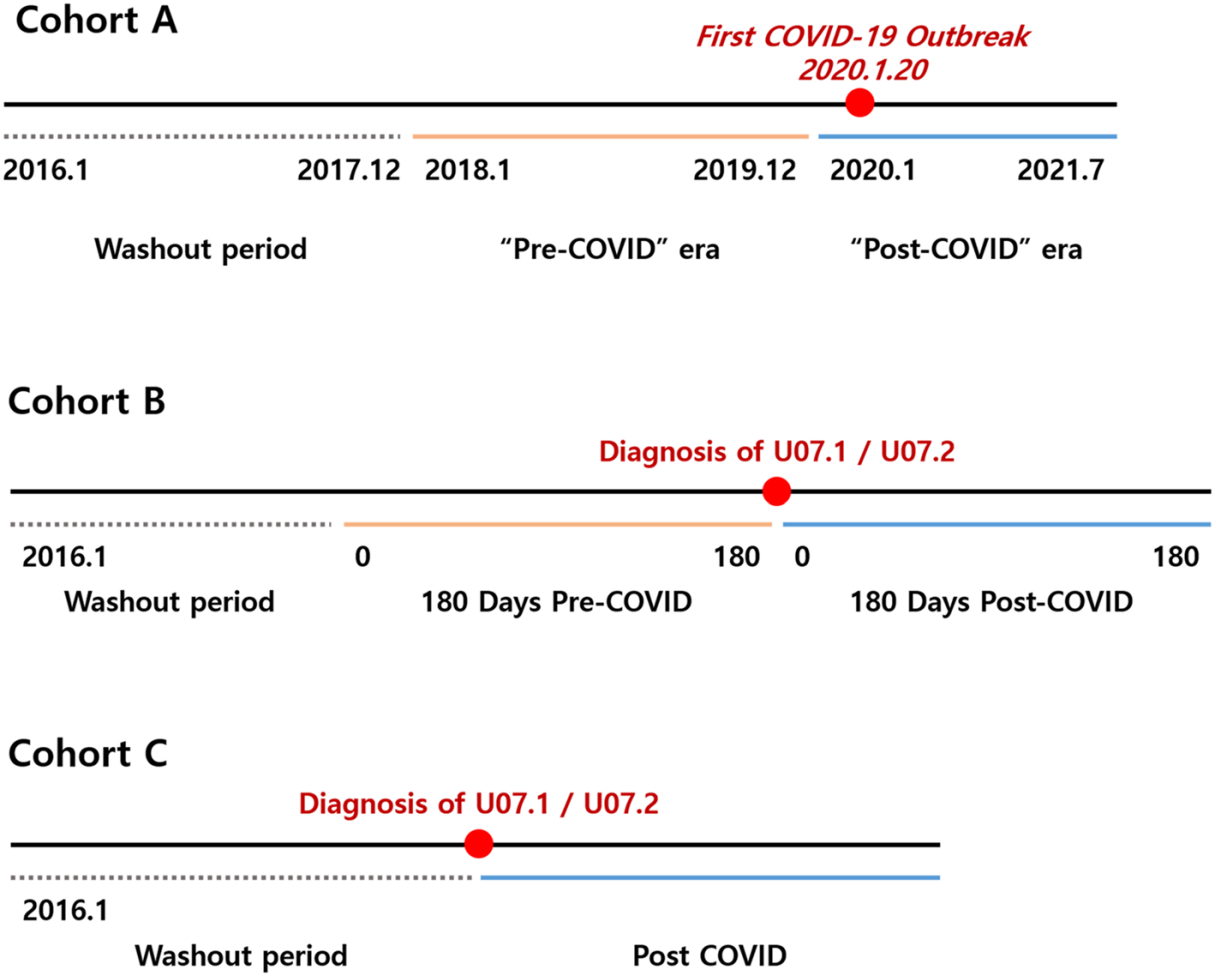
Schematic demonstration of periods and method for analyses. (**A**) Cohort A (**B**) Cohort B (**C**) Cohort C.

The incidence of retinal vascular occlusion before and after the COVID-19 outbreak was assessed using Cohort A, which included retinal vascular occlusion patients diagnosed with H34.1, H34.2, or H34.8 from January 2016 to July 2021. Steps taken to divide Cohort A according to time periods to exclude the washout period during 2016–2017 are demonstrated in Fig. 1A.

The numbers of new cases of RVO and RAO from 2018 to July 2021 by age group and year are shown in Supplementary Tables 1 and 2. A total of 52,664, 52,063, and 45,436 new cases of RVO occurred in 2018, 2019, and 2020, respectively, and 34,883 new cases of RVO occurred from January to July 2021. In 2018, there were 6,123 new cases of RAO, while the number was 5,861 and 6,094 for 2019 and 2020, respectively. Further, 3618 new RAO cases occurred from January to July 2021. Monthly new cases of RVO and RAO from January 2018 to July 2021 are presented in Supplementary Table 3.

The incidence rate of RVO and RAO by age group between 2018–2019 and 2020–2021 is demonstrated in Tables 1 and 2. The incidence in 2021 was calculated using data from diagnoses until July 2021. The population in the middle of the year was used as a reference for calculating the incidence rate, which is presented in Supplementary Table 4.

**Table 1:** Incidence rate of retinal vein occlusions in 2018–2019 and 2020–2021.

Age group	Total			Men			Women		
	2018–2019	2020–2021	p-value	2018–2019	2020–2021	p-value	2018–2019	2020–2021	p-value
0–4	0.94 (0.64–1.24)	0.26 (0.07–0.45)	< 0.001	0.84 (0.44–1.24)	0.22 (-0.03–0.46)	< 0.001	1.04 (0.59–1.50)	0.30 (0.01–0.60)	< 0.001
5–9	2.72 (2.25–3.20)	1.21 (0.86–1.57)	< 0.001	2.63 (1.98–3.28)	1.40 (0.86–1.94)	< 0.001	2.82 (2.13–3.51)	1.02 (0.55–1.49)	< 0.001
10–14	2.78 (2.30–3.26)	2.14 (1.67–2.62)	0.009	3.50 (2.75–4.25)	1.90 (1.28–2.52)	< 0.001	2.00 (1.42–2.59)	2.41 (1.69–3.13)	0.228
15–19	5.95 (5.31–6.59)	5.67 (4.93–6.42)	0.443	5.69 (4.83–6.56)	5.56 (4.54–6.58)	0.796	6.23 (5.28–7.17)	5.80 (4.71–6.88)	0.411
20–24	8.25 (7.56–8.93)	9.70 (8.85–10.55)	< 0.001	7.59 (6.69–8.50)	8.82 (7.70–9.95)	0.017	8.97 (7.94–10.01)	10.65 (9.36–11.95)	0.005
25–29	11.85 (11.03–12.67)	13.07 (12.12–14.02)	0.007	10.66 (9.59–11.73)	12.45 (11.17–13.72)	0.003	13.17 (11.91–14.42)	13.76 (12.34–15.18)	0.391
30–34	16.78 (15.77–17.78)	18.29 (17.10–19.48)	0.007	18.15 (16.70–19.61)	19.15 (17.47–20.84)	0.213	15.31 (13.93–16.69)	17.36 (15.69–19.03)	0.009
35–39	26.13 (25.01–27.25)	26.17 (24.86–27.48)	0.951	29.49 (27.82–31.16)	29.92 (27.96–31.87)	0.658	22.63 (21.14–24.12)	22.25 (20.53–23.98)	0.657
40–44	41.40 (39.97–42.82)	44.42 (42.75–46.08)	< 0.001	48.15 (45.99–50.31)	50.70 (48.20–53.20)	0.031	34.43 (32.58–36.29)	37.92 (35.73–40.12)	< 0.001
45–49	65.81 (64.14–67.49)	64.13 (62.23–66.03)	0.065	71.35 (68.90–73.80)	68.90 (66.14–71.66)	0.065	60.11 (57.82–62.39)	59.21 (56.62–61.81)	0.483
50–54	101.82 (99.66–103.98)	97.30 (94.98–99.63)	< 0.001	103.96 (100.89–107.02)	98.88 (95.58–102.18)	0.002	99.64 (96.61–102.67)	95.70 (92.43–98.98)	0.014
55–59	146.26 (143.69–148.82)	137.58 (134.75–140.40)	< 0.001	142.76 (139.18–146.34)	135.99 (132.03–139.95)	< 0.001	149.77 (146.09–153.44)	139.18 (135.14–143.22)	< 0.001
60–64	215.71 (212.26–219.15)	196.18 (192.67–199.68)	< 0.001	202.82 (198.06–207.58)	186.30 (181.44–191.16)	< 0.001	228.26 (223.28–233.24)	205.82 (200.77–210.87)	< 0.001
65–69	294.55 (289.69–299.41)	266.69 (261.81–271.56)	< 0.001	276.45 (269.66–283.23)	256.53 (249.65–263.41)	< 0.001	311.36 (304.42–318.30)	276.16 (269.27–283.05)	< 0.001
70–74	386.13 (379.81–392.44)	331.15 (324.86–337.45)	< 0.001	395.61 (386.20–405.01)	329.79 (320.60–338.98)	< 0.001	377.97 (369.45–386.50)	332.35 (323.71–340.99)	< 0.001
75–79	414.45 (407.38–421.51)	360.70 (353.30–368.09)	< 0.001	404.25 (393.48–415.02)	371.50 (360.03–382.98)	< 0.001	421.82 (412.46–431.18)	352.63 (342.97–362.29)	< 0.001
80–84	407.96 (399.21–416.71)	363.12 (354.29–371.95)	< 0.001	405.79 (391.30–420.27)	376.30 (361.61–390.99)	< 0.001	409.19 (398.21–420.18)	355.24 (344.20–366.28)	< 0.001
85–90	357.88 (345.92–369.85)	310.57 (299.07–322.08)	< 0.001	471.31 (445.60–497.02)	356.18 (333.71–378.65)	< 0.001	312.60 (299.38–325.83)	290.98 (277.67–304.29)	0.001
90–94	229.31 (212.77–245.84)	206.14 (190.11–222.17)	0.005	324.61 (282.84–366.38)	290.46 (250.30–330.61)	0.102	202.15 (184.55–219.74)	181.71 (164.62–198.81)	0.021
> = 90	143.16 (117.76–168.57)	114.84 (90.85–138.83)	0.024	317.30 (230.22–404.39)	253.21 (170.50–335.93)	0.139	102.68 (78.80–126.57)	83.32 (60.67–105.96)	0.103
Total	102.04 (101.42–102.65)	98.80 (98.12–99.48)	< 0.001	94.97 (94.13–95.81)	93.30 (92.36–94.24)	< 0.001	109.07 (108.17–109.97)	104.27 (103.28–105.27)	< 0.001

*The incidence of 2021 was calculated using data until July 2021.

**Table 2:** Incidence rate of retinal artery occlusions in 2018–2019 and 2020–2021.

Age group	Total			Men			Women		
	2018–2019	2020–2021	p-value	2018–2019	2020–2021	p-value	2018–2019	2020–2021	p-value
0–4	0.08 (− 0.01 to 0.16)	0.00 (0.00–0.00)	< 0.001	0.10 (− 0.04–0.24)	0.00 (0.00–0.00)	0.005	0.05 (− 0.05–0.15)	0.00 (0.00–0.00)	0.045
5–9	0.11 (0.01–0.20)	0.03 (− 0.03–0.08)	0.035	0.00 (0.00–0.00)	0.05 (− 0.05–0.16)	0.045	0.22 (0.03–0.41)	0.00 (0.00–0.00)	< 0.001
10–14	0.19 (0.07–0.32)	0.11 (0.00–0.21)	0.152	0.29 (0.08–0.51)	0.05 (− 0.05–0.16)	0.003	0.09 (− 0.03–0.21)	0.17 (− 0.02–0.36)	0.329
15–19	0.59 (0.39–0.79)	0.38 (0.19–0.57)	0.036	0.79 (0.47–1.11)	0.39 (0.12–0.66)	0.008	0.37 (0.14–0.60)	0.37 (0.10–0.64)	0.977
20–24	0.59 (0.41–0.77)	0.76 (0.52–1.00)	0.110	0.48 (0.25–0.70)	0.67 (0.36–0.98)	0.152	0.71 (0.42–1.01)	0.86 (0.49–1.22)	0.402
25–29	1.36 (1.08–1.63)	1.13 (0.85–1.41)	0.113	1.26 (0.89–1.63)	1.12 (0.74–1.51)	0.484	1.47 (1.05–1.89)	1.14 (0.73–1.55)	0.127
30–34	1.60 (1.29–1.91)	1.67 (1.31–2.03)	0.699	1.89 (1.42–2.35)	1.82 (1.30–2.34)	0.804	1.29 (0.89–1.70)	1.51 (1.01–2.00)	0.361
35–39	2.47 (2.12–2.81)	2.11 (1.74–2.48)	0.050	2.87 (2.35–3.39)	2.43 (1.87–2.99)	0.106	2.04 (1.59–2.49)	1.77 (1.29–2.26)	0.264
40–44	3.06 (2.67–3.45)	3.45 (2.98–3.91)	0.074	3.25 (2.69–3.81)	4.00 (3.30–4.70)	0.020	2.86 (2.32–3.39)	2.87 (2.27–3.48)	0.962
45–49	4.78 (4.33–5.24)	4.69 (4.18–5.20)	0.712	5.69 (4.99–6.38)	5.96 (5.14–6.77)	0.491	3.85 (3.28–4.43)	3.38 (2.76–4.00)	0.123
50–54	7.95 (7.35–8.55)	6.68 (6.08–7.29)	< 0.001	8.57 (7.69–9.45)	7.81 (6.88–8.73)	0.098	7.32 (6.50–8.14)	5.54 (4.76–6.33)	< 0.001
55–59	13.51 (12.73–14.29)	11.98 (11.15–12.82)	< 0.001	16.14 (14.93–17.34)	14.63 (13.33–15.93)	0.018	10.88 (9.88–11.87)	9.30 (8.25–10.34)	0.002
60–64	20.80 (19.73–21.87)	18.94 (17.85–20.02)	< 0.001	23.58 (21.96–25.20)	23.12 (21.41–24.83)	0.602	18.08 (16.68–19.48)	14.85 (13.49–16.20)	< 0.001
65–69	35.05 (33.38–36.73)	29.47 (27.85–31.09)	< 0.001	43.13 (40.45–45.81)	35.09 (32.55–37.63)	< 0.001	27.55 (25.48–29.61)	24.22 (22.18–26.26)	0.002
70–74	62.96 (60.41–65.52)	60.57 (57.88–63.26)	0.073	96.94 (92.28–101.59)	91.71 (86.87–96.56)	0.031	33.74 (31.19–36.28)	33.26 (30.53–35.99)	0.735
75–79	54.63 (52.07–57.20)	66.87 (63.69–70.05)	< 0.001	78.06 (73.32–82.79)	107.87 (101.69–114.05)	< 0.001	37.68 (34.88–40.48)	36.25 (33.15–39.35)	0.347
80–84	54.84 (51.63–58.05)	49.79 (46.52–53.06)	0.002	73.35 (67.19–79.51)	66.45 (60.28–72.62)	0.028	44.29 (40.68–47.90)	39.83 (36.13–43.53)	0.017
85–90	56.35 (51.60–61.10)	51.24 (46.57–55.92)	0.033	109.89 (97.47–122.30)	90.43 (79.11–101.75)	0.001	34.98 (30.55–39.40)	34.41 (29.83–38.99)	0.817
90–94	36.92 (30.29–43.56)	47.07 (39.41–54.73)	0.005	86.75 (65.16–108.34)	122.83 (96.72–148.94)	0.003	22.73 (16.83–28.63)	25.12 (18.76–31.48)	0.452
> = 90	19.95 (10.47–29.43)	31.32 (18.79–43.85)	0.043	49.77 (15.28–84.26)	105.50 (52.11–158.90)	0.013	13.02 (4.51–21.52)	14.42 (5.00–23.84)	0.771
Total	11.68 (11.47–11.89)	11.95 (11.71–12.18)	0.017	13.96 (13.64–14.29)	14.90 (14.52–15.27)	< 0.001	9.40 (9.13–9.66)	9.01 (8.72–9.30)	0.006

*The incidence of 2021 was calculated using data until July 2021.

The incidence rate per 100,000 people/year of RVO in 2018–2019 was 102.0 (95% CI 101.4–102.7) and was meaningfully lower in 2020–2021, with the incidence rate of 98.8 (98.1–99.5) (P =  < 0.001). The incidence rate of RAO in 2018–2019 was 11.7 (11.5–11.9) and was slightly higher in 2020–2021, with an incidence rate of 12.0 (11.7–12.2) (P = 0.017). The age group with the most significant incidence rate of RVO was 75–79 years in 2018–2019, with an incidence rate of 414.45 (407.38–421.51), while it was 80–84 years in 2020–2021, with an incidence rate of 363.12 (354.29–371.95). The age group with the highest incidence rate of RAO was 70–74 years in 2018–2019, with an incidence rate of 62.96 (60.41–65.52), while it was 75–79 years in 2020–2021, with an incidence rate of 66.98 (63.69–70.05).

### Incidence of retinal vascular occlusion before and after SARS-CoV-2 infection

The process of patient selection for Cohort B is presented in Supplementary Fig. 2. The number of patients diagnosed as U07.1 or U07.2 before January 31, 2021, was 104,090. Among them, 724 patients diagnosed as H34.1, H34.2, or H34.8 during 180 days before U07.1 or U07.2 diagnoses were excluded. Further, 42 patients diagnosed as H3.41, H34.2, or H34.8 during 180 days after the U07.1 or U07.2 diagnoses were excluded. Therefore, the number of patients in Cohort B diagnosed with U07.1 and U07.2 was 73,074 and 30,250, respectively.

The demographics of the 73,584 COVID-19 patients diagnosed with U07.1 and 30,506 suspected patients with the diagnosis of U07.2 reported until January 2021 are shown in Table 3. The mean age of patients diagnosed with U07.1 was 47.0 ± 21.3, while it was 45.48 ± 22.57 for patients diagnosed with U07.2, and a significant difference was noted in age composition between the two groups (P < 0.001, Chi-square test). Among the patients diagnosed with U07.1, 21,421 (29.1%) had DM and 24,109 (32.8%) had hypertension. Among those diagnosed with U07.2, 8,824 (28.9%) had DM and 9,913 (32.5%) had hypertension. No significant difference was observed in the rate of DM or hypertension between the U07.1 and U07.2 groups. The rate of CKD and stroke was higher in U07.2 patients than in U07.1 patients (CKD, 2713 [3.7%] vs. 2071 [6.8%], P < 0.001; stroke, 4355 [5.9%] vs. 2673 [8.8%], P < 0.001). In the U07.1 group, there were 35,079 (47.7%) urban residents while there were 38,505 (52.3%) rural residents and the number was 19,997 (65.6%) and 10,509 (34.4%), respectively, for the U07.2 group, with a significant difference in composition (P < 0.001).

**Table 3:** Demographics of the COVID-19 patients diagnosed with U07.1 and U07.2 reported until January 2021.

	U07.1	U07.2	p-value
Number (%)	73,584	30,506	
Age (years) Mean [SD]	47.00 [21.34]	45.48 [22.57]	
< 20	18,322 (24.9)	10,656 (34.9)	< 0.0001
20–39	30,210 (41.1)	9418 (30.9)	
40–64	8884 (12.1)	3400 (11.1)	
> = 65	16,168 (22.0)	7032 (23.1)	
Sex			
Male	35,652 (48.5)	16,316 (53.5)	< 0.0001
Female	37,932 (51.5)	14,190 (46.5)	
DM	21,421 (29.1)	8824 (28.9)	0.55
Hypertension	24,109 (32.8)	9913 (32.5)	0.40
Dyslipidemia	36,993 (50.3)	15,217 (49.9)	0.25
CKD	2713 (3.7)	2071 (6.8)	< 0.0001
Stroke	4355 (5.9)	2673 (8.8)	< 0.0001
Residence			
Urban	35,079 (47.7)	19,997 (65.6)	< 0.0001
Rural	38,505 (52.3)	10,509 (34.4)	

*DM* diabetes mellitus, *CKD* chronic kidney disease.

Table 4 presents the average incidence of RVOs and RAOs relative to the date of COVID-19 diagnosis using Cohort B. Analysis of Cohort B is schematically demonstrated in Fig. 1-B. In patients with U07.1, there were 28 (0.04%) new cases of RVOs in the 180 days pre-COVID diagnosis, while there were 45 (0.06%) new cases in the 180 days post-COVID diagnosis period. Further, 31 of the 45 (68.9%) newly reported RVO cases after COVID-19 diagnosis were reported 60 days after the diagnosis of U07.1. Meanwhile, there were 4 (0.01%) new RAO cases in the pre-COVID period, while there were 3 (< 0.01%) new RAO cases in the post-COVID diagnosis period. In the U07.2 group, 28 (0.09%) new RVOs occurred in the pre-COVID period, while 13 (0.04%) new cases occurred in the post-COVID period. Three (0.01%) new RAO cases developed in the U07.2 group before the diagnosis, while only one (< 0.01%) case occurred after the diagnosis. In the U07.1 group, the unadjusted IRR of RVOs during the 180 days post-COVID diagnosis was 1.60 (1.00–2.58), compared to the reference incidence (1.00) of pre-COVID diagnosis (P = 0.049). After adjustment for age and sex, the difference narrowed with an adjusted IRR of 1.03 (0.62–1.70) with no significant difference (P = 0.906). The unadjusted IRR of RAOs in the U07.1 group during the 180 days post-COVID diagnosis was 1.25 (0.34–4.66), compared to pre-COVID diagnosis, and the adjusted IRR was 1.00 (0.06–15.99), with no significant difference for both (P = 0.739 and 1.000, respectively). In the U07.2 group, the unadjusted IRR of RVOs in the post-COVID period was 0.46 (0.24–0.90) and was significantly lower in post-COVID diagnosis (P = 0.022); however, after adjustment, the IRR was 0.70 (0.33–1.49) and showed no significant difference (P = 0.359). The unadjusted IRR of RAO in the post-COVID period was 0.33 (0.03–3.21), and the adjusted IRR was 26.34 (0.52–1327.42) with no significant difference (P = 0.341 and 0.102, respectively).

**Table 4:** Average incidence of retinal vascular occlusions relative to the date of COVID-19 diagnosis.

	Days	1–20	21–40	41–60	61–80	81–100	101–120	121–140	141–160	161–180	Total number (1–180)	Crude rate per 1 million person-month	Unadjusted IRR (95% CI)	P-value	Adjusted IRR (95% CI)	P-value
U071, RVO	PreCOVID	2	7	4	2	3	3	2	2	3	28	65.454	1 (Reference)	–	1 (Reference)	–
	PostCOVID	4	6	4	9	1	4	5	4	8	45	105.112	1.607 (1.003–2.576)	0.049	1.031 (0.622–1.708)	0.906
U071, RAO	PreCOVID	0	0	2	0	0	1	1	0	0	4	9.344	1 (Reference)	–	1 (Reference)	–
	PostCOVID	1	0	0	1	0	1	0	1	1	5	11.680	1.250 (0.336–4.655)	0.739	1.000 (0.063–15.988)	1.000
U072, RVO	PreCOVID	5	4	1	0	0	2	6	6	4	28	158.618	1 (Reference)	–	1 (Reference)	–
	PostCOVID	2	0	2	1	1	1	3	2	1	13	73.644	0.464 (0.240–0.896)	0.022	0.704 (0.333–1.488)	0.359
U072, RAO	PreCOVID	0	1	1	0	0	0	0	1	0	3	16.994	1 (Reference)	–	1 (Reference)	–
	PostCOVID	0	0	0	0	0	0	0	0	1	1	5.664	0.333 (0.035–3.205)	0.341	26.339 (0.523–1327.417)	0.102

*RVO* retinal vein occlusion, *RAO* retinal artery occlusion, *COVID* the coronavirus disease, *IRR* incidence rate ratio.

In the sub-analysis of severe COVID-19 patients, there was no significant difference in unadjusted and adjusted IRR for RAO and RVO occurrence in both U07.1 and U07.2 patient groups (Supplementary Table 5). The sub-analysis for coagulopathy patients could not be conducted because of the scarcity of patients diagnosed with RAO or RVO in the cohort. There were two cases of RVO and no case of RAO in the pre-COVID period, and no post-COVID diagnosis of RAO or RVO in this subgroup.

### Comparison of incidence of retinal vascular occlusion between COVID-19 patients and the general population

The process of patient selection for Cohort C is demonstrated in Supplementary Fig. 3. Among 104,090 patients diagnosed as either U07.1 or U07.2 before January 31, 2021, 786 patients diagnosed as H34.1, H34.2, or H34.8 were excluded. The number of patients in Cohort C diagnosed with U07.1 was 73,070, while that of U07.2 was 30,234.

A comparison of the incidence of RVOs and RAOs in COVID-19 patients after the diagnosis of COVID-19 with the incidence of RVOs and RAOs in the general population (between 2018–2019 and 2020–2021) using Cohort C was made. Analysis of Cohort C is schematically demonstrated in Fig. 1-C. In patients with a diagnosis of U07.1, the SIR of RVOs was 1.00 (0.78–1.25), with no significant difference (P = 0.988) using the 2018–2019 period as a reference. Based on the incidence during 2020–2021, the SIR was 1.10 (0.86–1.38), with no significant difference. (P = 0.443) The SIR of RAOs in the U07.1 patient group was 0.96 (0.45–1.82) based on 2018–2019 incidence, and it was 1.00 (0.46–1.90) based on 2020–2021 incidence; there was no significant difference (P = 0.950 and 0.953, respectively). In patients with a diagnosis of U07.2, the SIR of RVOs was 0.87 (0.59–1.24) using the incidence during the 2018–2019 period as a reference and 0.96 (0.65–1.36) using the incidence during 2020–2021 (P = 0.46 and 0.84, respectively). The SIR of RAOs in the U07.2 group was 0.46 (0.08–1.52) during 2018–2019 and 0.47 (0.08–1.55) during 2020–2021 and showed no clinically significant difference (P = 0.26 and 0.27, respectively).

In the sub-analysis of severe COVID-19 patients, no significant increase was observed in the SIR of RAO and RVO in both U07.1 and U07.2 patient groups, using the 2018–2019 period as a reference. In patients with a diagnosis of U07.1, the SIR of RVOs was 0.59 (0.32–1.00), using the 2018–2019 period as a reference (P = 0.049). Based on the incidence during 2020–2021, the SIR of RVOs was 0.66 (0.36–1.12), with no significant difference (P = 0.134). The SIR of RAOs in the U07.1 patient group was 0.70 (0.12–2.32) based on 2018–2019 incidence, and it was 0.71 (0.12–2.35) based on 2020–2021 incidence; no significant difference was noted (P = 0.682 and 0.694, respectively). In patients with a diagnosis of U07.2, the SIR of RVOs was 1.20 (0.67–2.00), using the incidence during the 2018–2019 period as a reference and 1.36 (0.75–2.26), using the incidence during 2020–2021 (P = 0.50 and 0.28, respectively). The SIR of RAOs in the U07.2 group was 1.18 (0.20–3.91) during 2018–2019 and 1.17 (0.20–3.87) during 2020–2021 and showed no clinically significant difference (P = 0.75 and 0.75, respectively). The sub-analysis for coagulopathy patients could not be conducted because of the small number of patients diagnosed with RAO or RVO in the cohort.

## Discussion

Our study showed that the incidence of RVO was slightly higher during 180 days after the diagnosis of COVID-19 compared to that during 180 days before the diagnosis. However, after accounting for confounders such as age and sex, no significant correlation was observed between RVO and SARS-CoV-2 infection. The incidence of RAO was slightly higher in the 2020–2021 period than in 2018–2019, but no significant correlation between RAO and SARS-CoV-2 infection was demonstrated in any of the analyses.

As the Korean national health insurance data accounts for nearly 97% of the Korean population, this data is of representative value on incidence rates of RVOs and RAOs in COVID-19 in South Korea. As of January 31, 2021, a total of 73,584 patients were diagnosed with U07.1 according to the national health insurance data, comprising 94.1% of the entire 78,197 COVID-19 patients confirmed by Statistics Korea. Furthermore, considering that only some foreign population does not benefit from Korean health insurance services, this number shows that health insurance data can represent most of the Korean population with credibility.

After ocular manifestations related to COVID-19 were reported, studies have focused on finding out whether the hypercoagulable state caused by COVID-19 can cause retinal vascular abnormality, with RVO and RAO being the most common^16^. However, although many case reports and series have reported suspected RVOs and RAOs caused by COVID-19 based on temporal relationships, the evidence for a definite correlation is yet to be found^7–15, 17^. Some reports that led to the conclusion of COVID-19 as a cause or at least as a factor leading to retinal vascular complications were cases with no typical risk factors, such as old age or comorbidities, including DM or hypertension, and some bilateral cases^7, 10, 11, 13, 14, 18^.

As no definite pathophysiology led to the association of COVID-19 with retinal vascular diseases, other attempts were made to elucidate the correlation between the two. The only study on changes in the incidence of retinal vascular occlusions after COVID-19 diagnosis before this study was based on cohort data of over 430,000 patients from an integrated health care organization based in a particular region; the study concluded that there was an increase in the incidence of RVOs (adjusted IRR, 1.54; 95% CI 1.05–2.26; P = 0.03) and no change in the incidence of RAOs after SARS-CoV-2 infection^19^. Similar to our results, there was no change in RAO incidence after the diagnosis of COVID-19; however, there was no change in RVO incidence as well after adjustment of age and sex in our study. The study's authors also explained that these events remained rare, and a cause-and-effect relationship could not be made due to the absence of randomized controls^19^. Another study focused on the incidence of RAO and RVO in patients visiting retina clinics during the COVID-19 pandemic. It concluded that the percentage of new cases of RAO and RVO with respect to all new diagnoses remained stable for most of the COVID-19 period^20^. This study pointed out that there had been an increase in the percentages of central RAO, central RVO, and branch RVO of all new diagnoses during the first few months of the COVID-19 period, which may have led to the presumption that more patients presented with these conditions during COVID-19^20^.

In this study, the incidence of RVOs after the diagnosis of COVID-19 was slightly higher during 180 days after the diagnosis. However, 31/45 (68.9%) of these newly reported cases were reported after 60 days of the diagnosis of COVID-19. According to a review article on central RVO secondary to COVID-19, symptom onset ranged from 5 days to 6 weeks after the initial complaint of fever^18^. Although the authors set a wide margin of 180 days after the diagnosis of COVID-19 to include possible delayed reports of ocular symptoms, because focus is generally on systemic symptoms as a result of COVID-19, the study results suggest that many of the RVO cases reported after 42 days of COVID-19 diagnoses might not be directly associated with COVID-19 infection.

This study included "suspected" COVID-19 patients with the diagnosis of U07.2 and confirmed patients with the diagnosis of U07.1 for comparison of the two groups. The group of suspected patients seems to have been comprised of real COVID-19 patients with unclear laboratory results and some non-COVID-19 patients with respiratory symptoms. Although some aspects of this group differed from that of the confirmed COVID-19 patients, as seen in Table 3, the fact that all analyses with the U07.2 group revealed no distinct correlation between retinal vascular occlusions and COVID-19 adds plausibility to the possibility that COVID-19 might not have a significant influence on the incidence of retinal vascular occlusions.

Strengths of this study include its large size with a relatively long follow-up duration which could add up to its credibility. Limitations of the study include the retrospective study design with restricted data that did not allow us to add covariates that might have influenced the development of retinal vascular occlusions and their reporting date, such as the date and duration of hospitalization. Additionally, factors such as nutritional and smoking statuses and population movement that may have influenced the results could not be taken into account in the analyses. As the study population included patients before the large-scale vaccination for COVID-19 began in South Korea in February 2021, the effect of vaccination on the occurrence of RAO and RVO could not be assessed. As the Delta variant (B.1.617) of COVID-19 was first reported in South Korea in April 2021, after the time interval in which the study population of this study was analyzed, the cohort most likely was affected by the wild type and Alpha variant (B1.1.7) of COVID-19. Therefore, the association of the different variants of COVID-19 with the development of RAO and RVO could not be evaluated.

In conclusion, our study did not find any definite correlation between retinal vascular occlusions and SARS-CoV-2 infection. Therefore, further studies with a possibly larger population and prospective cohorts are required to clarify the association between the two.

### Supplementary Information

Supplementary Information.

## Data Availability

The data that support the findings of this study are available from the Healthcare Bigdata Hub of Health Insurance Review & Assessment Service of Korea (https://opendata.hira.or.kr/). However, restrictions apply to the availability of these data, which were used under license for the current study, and as a result, they are not publicly available. Data are however available from the authors upon reasonable request and with the permission of the Health Insurance Review & Assessment Service of Korea. Some datasets are available upon reasonable request from the corresponding author, Yong Joon Kim.
