# Supplementary material for: Retinal vascular occlusion risks during the COVID-19 pandemic and after SARS-CoV-2 infection
Source: Sci Rep. 2023 Oct 6;13:16851. doi: 10.1038/s41598-023-44199-z (PMC10558568; doi:10.1038/s41598-023-44199-z)
Supplement: Supplementary file 1 — Supplementary Information. [file 41598_2023_44199_MOESM1_ESM.docx]

**Supplementary Materials for:**

**Retinal Vascular Occlusion Risks**

**during the COVID-19 Pandemic and after SARS-CoV-2 Infection**

Hyo Song Park^1,2^, Sunyeop Kim^3^, Christopher Seungkyu Lee^4^, Suk Ho Byeon^4^, Sung Soo Kim^4^, Seung Won Lee^5*^, and Yong Joon Kim^4*^

^1^Department of Ophthalmology, College of Medicine, Soonchunhyang University, Cheonan, Korea

^2^Department of Ophthalmology, Soonchunhyang University Hospital Bucheon, Bucheon, Korea

^3^Department of Medical AI, Sungkyunkwan University School of Medicine, Suwon, Korea

^4^The Institute of Vision Research, Department of Ophthalmology, Yonsei University College of Medicine, Seoul, Korea

^5^Department of Precision Medicine, Sungkyunkwan University School of Medicine, Suwon, Korea

*****Seung Won Lee and Yong Joon Kim contributed equally to this manuscript and are considered co-corresponding authors.

**Supplementary Figures 1-3**

**Supplementary Tables 1-5**

**Supplementary Figure 1.** Monthly confirmed COVID-19 cases in South Korea from January 2021 to January 2022


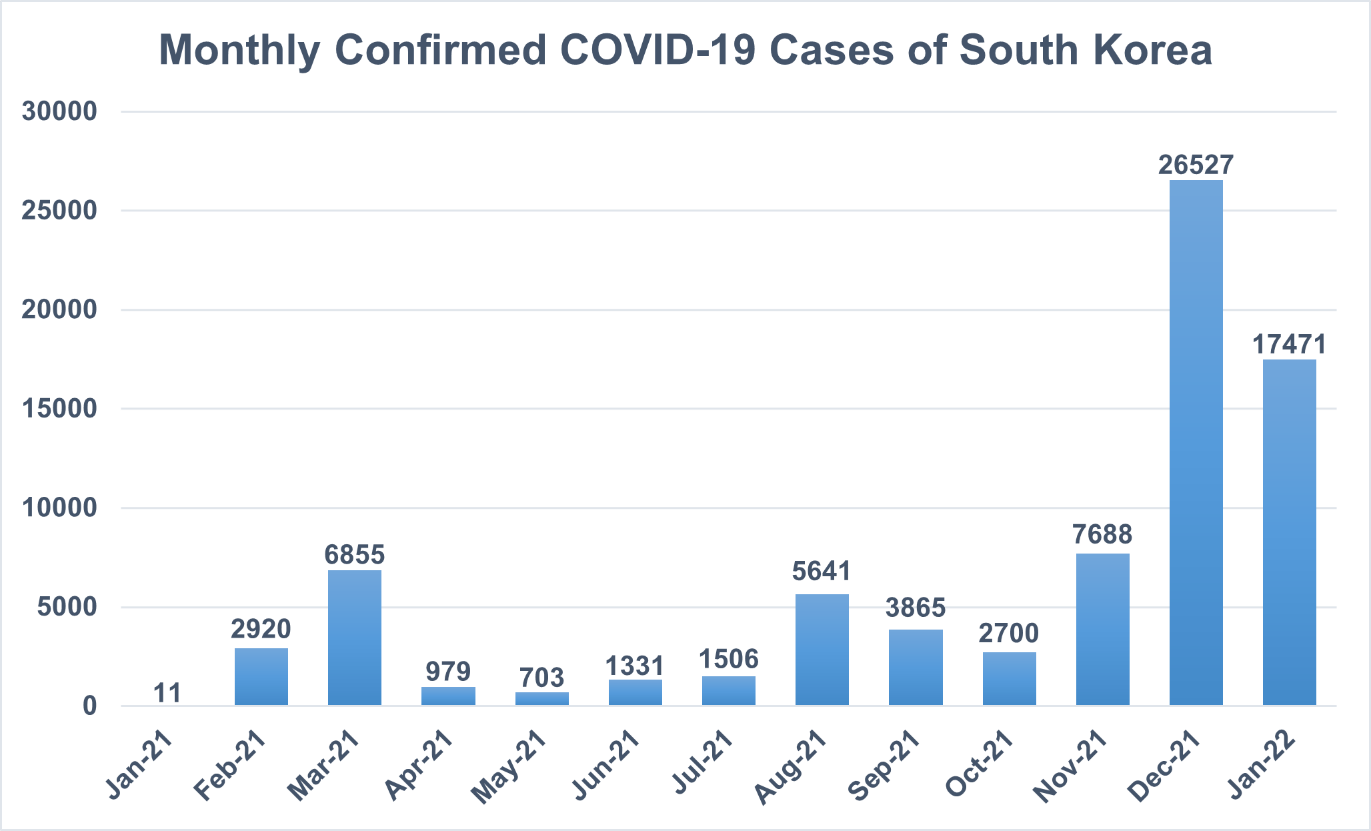


**Supplementary Figure 2.** Schematic demonstration of the steps for patient selection in Cohort B.


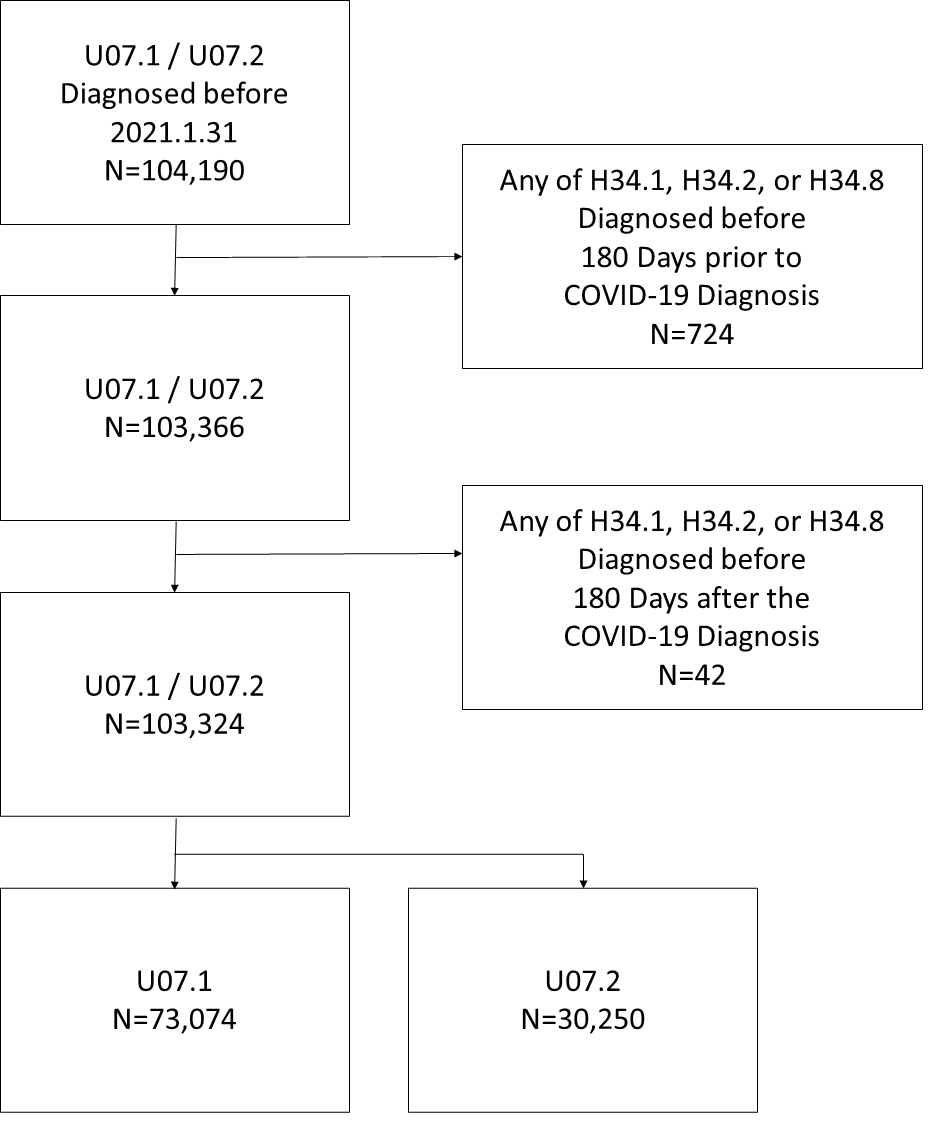


**Supplementary Figure 3.** Schematic demonstration of the steps for patient selection in Cohort C.


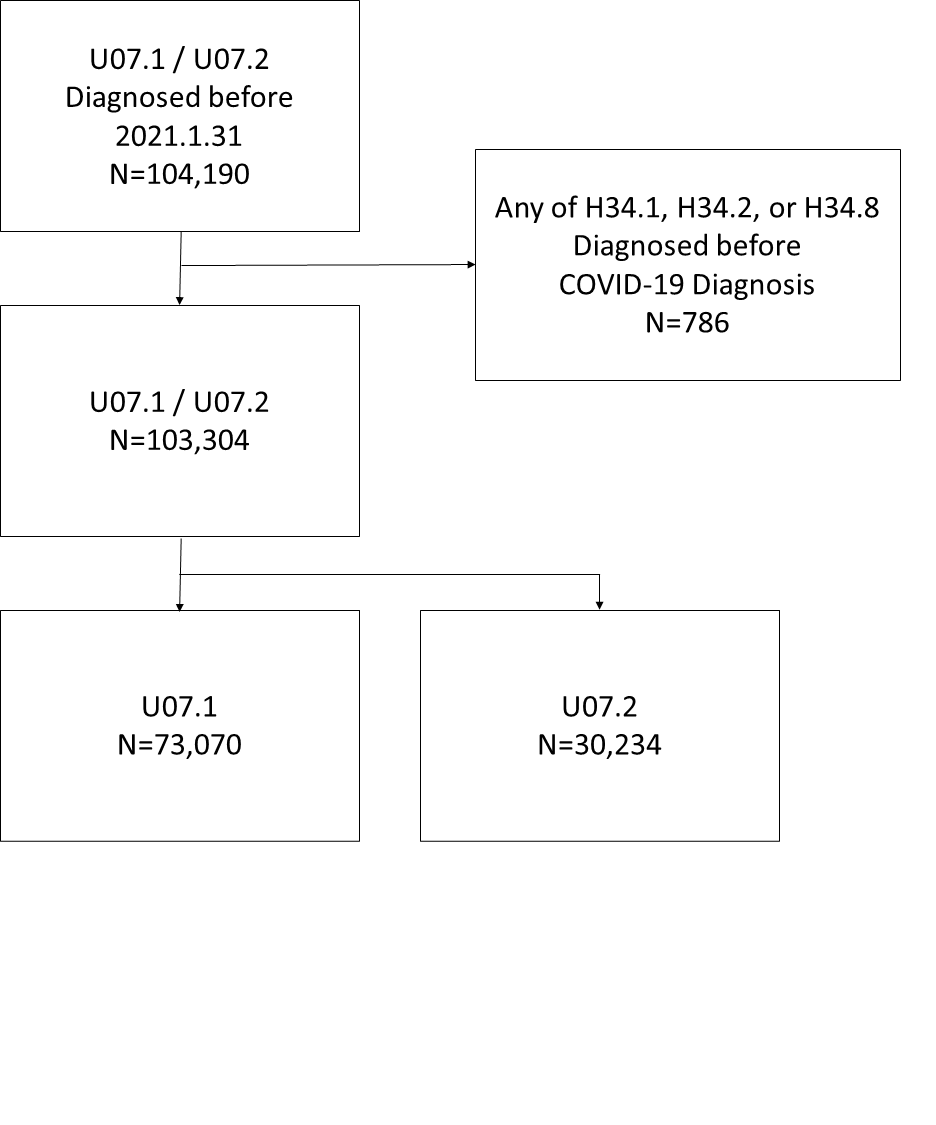


**Supplementary Table 1.** New cases of retinal vein occlusions from 2018 to July 2021 by age group and year.

|  | 2018 | | | 2019 | | | 2020 | | | 2021 | | |
| --- | --- | --- | --- | --- | --- | --- | --- | --- | --- | --- | --- | --- |
| Age group | Total | Men | Women | Total | Men | Women | Total | Men | Women | Total | Men | Women |
| 0–4 | 14 | 7 | 7 | 23 | 10 | 13 | 4 | 2 | 2 | 3 | 1 | 2 |
| 5–9 | 43 | 28 | 15 | 84 | 35 | 49 | 20 | 13 | 7 | 24 | 13 | 11 |
| 10–14 | 54 | 39 | 15 | 75 | 45 | 30 | 39 | 16 | 23 | 40 | 20 | 20 |
| 15–19 | 173 | 82 | 91 | 160 | 84 | 76 | 135 | 74 | 61 | 89 | 40 | 49 |
| 20–24 | 246 | 116 | 130 | 314 | 155 | 159 | 287 | 139 | 148 | 210 | 97 | 113 |
| 25–29 | 388 | 179 | 209 | 415 | 202 | 213 | 435 | 226 | 209 | 292 | 140 | 152 |
| 30–34 | 525 | 294 | 231 | 545 | 303 | 242 | 516 | 263 | 253 | 394 | 232 | 162 |
| 35–39 | 1098 | 606 | 492 | 990 | 595 | 395 | 975 | 587 | 388 | 564 | 312 | 252 |
| 40–44 | 1644 | 971 | 673 | 1592 | 940 | 652 | 1549 | 916 | 633 | 1184 | 669 | 515 |
| 45–49 | 3083 | 1661 | 1422 | 2847 | 1602 | 1245 | 2588 | 1434 | 1154 | 1803 | 961 | 842 |
| 50–54 | 4422 | 2217 | 2205 | 4145 | 2199 | 1946 | 3889 | 2055 | 1834 | 2836 | 1390 | 1446 |
| 55–59 | 6525 | 3172 | 3353 | 5955 | 2932 | 3023 | 5458 | 2735 | 2723 | 3636 | 1793 | 1843 |
| 60–64 | 7527 | 3475 | 4052 | 7524 | 3509 | 4015 | 6904 | 3274 | 3630 | 5135 | 2374 | 2761 |
| 65–69 | 7056 | 3194 | 3862 | 7052 | 3183 | 3869 | 6478 | 3080 | 3398 | 5034 | 2264 | 2770 |
| 70–74 | 6943 | 3061 | 3882 | 7407 | 3738 | 3669 | 5706 | 2611 | 3095 | 4928 | 2337 | 2591 |
| 75–79 | 6720 | 2579 | 4141 | 6495 | 2833 | 3662 | 5048 | 2078 | 2970 | 4095 | 1948 | 2147 |
| 80–84 | 4158 | 1471 | 2687 | 4188 | 1544 | 2644 | 3557 | 1409 | 2148 | 2941 | 1111 | 1830 |
| 85–90 | 1648 | 583 | 1065 | 1788 | 708 | 1080 | 1451 | 503 | 948 | 1349 | 462 | 887 |
| 90–94 | 339 | 99 | 240 | 400 | 133 | 267 | 347 | 106 | 241 | 288 | 95 | 193 |
| >=90 | 58 | 25 | 33 | 64 | 26 | 38 | 50 | 18 | 32 | 38 | 18 | 20 |
| Total | 52664 | 23859 | 28805 | 52063 | 24776 | 27287 | 45436 | 21539 | 23897 | 34883 | 16277 | 18606 |

**Supplementary Table 2.** New cases of retinal artery occlusions from 2018 to July 2021 by age group and year.

|  | 2018 | | | 2019 | | | 2020 | | | 2021 | | |
| --- | --- | --- | --- | --- | --- | --- | --- | --- | --- | --- | --- | --- |
| Age group | Total | Men | Women | Total | Men | Women | Total | Men | Women | Total | Men | Women |
| 0–4 | 0 | 0 | 0 | 3 | 2 | 1 | 0 | 0 | 0 | 0 | 0 | 0 |
| 5–9 | 3 | 0 | 3 | 2 | 0 | 2 | 1 | 1 | 0 | 0 | 0 | 0 |
| 10–14 | 5 | 4 | 1 | 4 | 3 | 1 | 2 | 0 | 2 | 2 | 1 | 1 |
| 15–19 | 16 | 13 | 3 | 17 | 10 | 7 | 8 | 3 | 5 | 7 | 5 | 2 |
| 20–24 | 21 | 10 | 11 | 19 | 7 | 12 | 18 | 8 | 10 | 21 | 10 | 11 |
| 25–29 | 45 | 23 | 22 | 47 | 22 | 25 | 34 | 16 | 18 | 29 | 17 | 12 |
| 30–34 | 49 | 31 | 18 | 53 | 31 | 22 | 49 | 26 | 23 | 34 | 21 | 13 |
| 35–39 | 100 | 54 | 46 | 97 | 63 | 34 | 87 | 49 | 38 | 37 | 24 | 13 |
| 40–44 | 116 | 57 | 59 | 123 | 72 | 51 | 126 | 75 | 51 | 86 | 50 | 36 |
| 45–49 | 215 | 130 | 85 | 216 | 130 | 86 | 213 | 137 | 76 | 108 | 70 | 38 |
| 50–54 | 322 | 174 | 148 | 347 | 190 | 157 | 276 | 164 | 112 | 186 | 108 | 78 |
| 55–59 | 564 | 348 | 216 | 589 | 342 | 247 | 532 | 331 | 201 | 260 | 156 | 104 |
| 60–64 | 709 | 396 | 313 | 742 | 416 | 326 | 726 | 444 | 282 | 436 | 257 | 179 |
| 65–69 | 880 | 528 | 352 | 799 | 467 | 332 | 810 | 449 | 361 | 462 | 282 | 180 |
| 70–74 | 1261 | 932 | 329 | 1079 | 734 | 345 | 1276 | 918 | 358 | 669 | 458 | 211 |
| 75–79 | 894 | 536 | 358 | 848 | 509 | 339 | 1028 | 693 | 335 | 667 | 476 | 191 |
| 80–84 | 564 | 262 | 302 | 558 | 283 | 275 | 534 | 258 | 276 | 357 | 187 | 170 |
| 85–90 | 290 | 175 | 115 | 251 | 126 | 125 | 278 | 155 | 123 | 184 | 90 | 94 |
| 90–94 | 60 | 36 | 24 | 59 | 26 | 33 | 83 | 46 | 37 | 62 | 39 | 23 |
| >=90 | 9 | 4 | 5 | 8 | 4 | 4 | 13 | 8 | 5 | 11 | 7 | 4 |
| Total | 6123 | 3713 | 2410 | 5861 | 3437 | 2424 | 6094 | 3781 | 2313 | 3618 | 2258 | 1360 |

**Supplementary Table 3.** Monthly new cases of retinal vein occlusion and retinal artery occlusion from January 2018 to July 2021.

A. Retinal vein occlusion

| Month  Year | 1 | 2 | 3 | 4 | 5 | 6 | 7 | 8 | 9 | 10 | 11 | 12 |
| --- | --- | --- | --- | --- | --- | --- | --- | --- | --- | --- | --- | --- |
| 2018 | 4280 | 3853 | 4996 | 4741 | 4356 | 4650 | 4839 | 4360 | 3969 | 4729 | 4048 | 3843 |
| 2019 | 4266 | 4233 | 4370 | 4514 | 4548 | 3937 | 5180 | 4588 | 4305 | 4134 | 3872 | 4116 |
| 2020 | 4125 | 3438 | 3383 | 3664 | 4149 | 4110 | 4210 | 3463 | 3673 | 3854 | 3615 | 3752 |
| 2021 | 5063 | 4229 | 6011 | 5052 | 4642 | 4950 | 4936 | - | - | - | - | - |

B. Retinal artery occlusion

| Month  Year | 1 | 2 | 3 | 4 | 5 | 6 | 7 | 8 | 9 | 10 | 11 | 12 |
| --- | --- | --- | --- | --- | --- | --- | --- | --- | --- | --- | --- | --- |
| 2018 | 451 | 448 | 501 | 515 | 521 | 503 | 580 | 561 | 413 | 551 | 519 | 560 |
| 2019 | 589 | 450 | 451 | 502 | 532 | 483 | 521 | 475 | 432 | 517 | 451 | 458 |
| 2020 | 534 | 461 | 462 | 491 | 495 | 511 | 566 | 554 | 506 | 484 | 496 | 534 |
| 2021 | 590 | 526 | 567 | 502 | 437 | 476 | 520 | - | - | - | - | - |

**Supplementary Table 4.** South Korea’s population in the middle of the year from 2018 to 2021

|  | 2018 | | | 2019 | | | 2020 | | | 2021 | | |
| --- | --- | --- | --- | --- | --- | --- | --- | --- | --- | --- | --- | --- |
| Age, group | Total | Men | Women | Total | Men | Women | Total | Men | Women | Total | Men | Women |
| 0–4 | 2,026,168.5 | 1,039,018 | 987,150.5 | 1,909,182.5 | 979,211 | 929,971.5 | 1,760,637.5 | 903,178 | 857,459.5 | 1,603,407.5 | 822,670.5 | 780,737 |
| 5–9 | 2,340,328 | 1,203,026 | 1,137,302 | 2,323,178.5 | 1,193,014 | 1,130,164.5 | 2,305,388.5 | 1,182,550.5 | 1,122,838 | 2,259,652.5 | 1,158,010.5 | 1,101,642 |
| 10–14 | 2,334,187 | 1,206,456 | 1,127,731 | 2,308,928.5 | 1,191,290.5 | 1,117,638 | 2,314,984 | 1,192,976.5 | 1,122,007.5 | 2,347,218 | 1,208,122.5 | 1,139,095.5 |
| 15–19 | 2,872,026 | 1,497,710 | 1,374,316 | 2,725,294.5 | 1,418,938 | 1,306,356.5 | 2,551,012.5 | 1,325,906 | 1,225,106.5 | 2,395,066 | 1,242,509 | 1,152,557 |
| 20–24 | 3,436,410.5 | 1,810,584.5 | 1,625,826 | 3,353,801 | 1,759,184 | 1,594,617 | 3,272,265 | 1,709,726 | 1,562,539 | 3,175,015 | 1,654,306.5 | 1,520,708.5 |
| 25–29 | 3,347,964.5 | 1,762,115 | 1,585,849.5 | 3,430,877 | 1,811,314.5 | 1,619,562.5 | 3,505,179.5 | 1,852,731 | 1,652,448.5 | 3,529,975 | 1,864,933.5 | 1,665,041.5 |
| 30–34 | 3,227,142.5 | 1,661,315 | 1,565,827.5 | 3,151,167 | 1,627,594 | 1,523,573 | 3,130,579 | 1,623,891.5 | 1,506,687.5 | 3,162,503.5 | 1,646,546.5 | 1,515,957 |
| 35–39 | 4,031,400 | 2,054,181 | 1,977,219 | 3,960,321 | 2,018,361.5 | 1,941,959.5 | 3,786,437.5 | 1,933,535.5 | 1,852,902 | 3,590,685.5 | 1,836,896 | 1,753,789.5 |
| 40–44 | 3,959,875 | 2,009,430 | 1,950,445 | 3,857,129 | 1,959,709 | 1,897,420 | 3,863,754.5 | 1,962,337 | 1,901,417.5 | 3,924,880 | 1,995,164 | 1,929,716 |
| 45–49 | 4,531,587.5 | 2,299,107 | 2,232,480.5 | 4,478,729.5 | 2,274,031.5 | 2,204,698 | 4,380,758 | 2,224,690 | 2,156,068 | 4,227,626.5 | 2,145,192 | 2,082,434.5 |
| 50–54 | 4,155,086 | 2,100,952.5 | 2,054,133.5 | 4,258,855 | 2,146,978 | 2,111,877 | 4,331,294 | 2,184,220 | 2,147,074 | 4,422,823 | 2,228,227 | 2,194,596 |
| 55–59 | 4,273,105 | 2,138,816.5 | 2,134,288.5 | 4,259,891.5 | 2,136,895 | 2,122,996.5 | 4,207,585 | 2,115,455 | 2,092,130 | 4,118,758.5 | 2,081,329.5 | 2,037,429 |
| 60–64 | 3,376,125.5 | 1,665,402.5 | 1,710,723 | 3,601,453 | 1,778,062.5 | 1,823,390.5 | 3,804,709.5 | 1,878,929.5 | 1,925,780 | 3,997,811.5 | 1,976,075 | 2,021,736.5 |
| 65–69 | 2,345,864.5 | 1,128,819 | 1,217,045.5 | 2,443,839.5 | 1,177,940 | 1,265,899.5 | 2,635,592 | 1,271,779.5 | 1,363,812.5 | 2,881,870 | 1,390,963.5 | 1,490,906.5 |
| 70–74 | 1,814,165 | 834,873.5 | 979,291.5 | 1,902,232 | 883,751 | 1,018,481 | 2,000,708.5 | 934,279.5 | 1,066,429 | 2,075,136.5 | 970,430 | 1,104,706.5 |
| 75–79 | 1,585,356 | 662,915.5 | 922,440.5 | 1,603,245.5 | 675,845 | 927,400.5 | 1,602,662.5 | 682,114.5 | 920,548 | 1,597,970 | 688,442 | 909,528 |
| 80–84 | 989,634.5 | 356,227 | 633,407.5 | 1,056,165 | 386,770.5 | 669,394.5 | 1,110,911.5 | 413,379.5 | 697,532 | 1,163,257.5 | 439,372 | 723,885.5 |
| 85–89 | 460,471.5 | 129,511.5 | 330,960 | 499,619 | 144,408.5 | 355,210.5 | 549,650.5 | 163,454 | 386,196.5 | 603,274.5 | 184,245.5 | 419,029 |
| 90–94 | 153,059.5 | 33,835 | 119,224.5 | 169,218.5 | 37,636 | 131,582.5 | 188,043.5 | 42,101.5 | 145,942 | 205,711 | 46,457.5 | 159,253.5 |
| >=95 | 40,923 | 7,665.5 | 33,257.0 | 44,296.0 | 8,408 | 35,888.5 | 47,106.0 | 8,846 | 38,260.5 | 50,611 | 9,209 | 41,402 |

**Supplementary Table 5.** Average incidence of retinal vascular occlusions relative to the date of COVID-19 diagnosis in severe COVID-19 patients

|  | Days | 1–20 | 21–40 | 41–60 | 61–80 | 81–100 | 101–120 | 121–140 | 141–160 | 161–180 | Total number (1-180) | Crude rate per 1 million person-month | Unadjusted IRR (95% CI) | P-value | Adjusted IRR (95% CI) | P-value |
| --- | --- | --- | --- | --- | --- | --- | --- | --- | --- | --- | --- | --- | --- | --- | --- | --- |
| U071, RVO | PreCOVID | 1 | 2 | 2 | 0 | 1 | 3 | 1 | 1 | 1 | 12 | 183.645 | 1  (Reference) | - | 1  (Reference) | - |
|  | PostCOVID | 1 | 0 | 1 | 1 | 0 | 0 | 1 | 1 | 1 | 6 | 103.705 | 0.565  (0.212-1.505) | 0.253 | 0.594  (0.222-1.592) | 0.300 |
| U071, RAO | PreCOVID | 0 | 0 | 0 | 0 | 0 | 1 | 0 | 0 | 0 | 1 | 15.304 | 1  (Reference) | - | 1  (Reference) | - |
|  | PostCOVID | 0 | 0 | 0 | 0 | 0 | 1 | 0 | 0 | 0 | 1 | 17.284 | 1.129  (0.071-18.056) | 0.931 | 1.195  (0.076-18.691) | 0.899 |
| U072, RVO | PreCOVID | 0 | 0 | 0 | 0 | 0 | 1 | 2 | 2 | 0 | 5 | 200.204 | 1  (Reference) | - | 1  (Reference) | - |
|  | PostCOVID | 2 | 0 | 0 | 1 | 0 | 0 | 1 | 1 | 1 | 6 | 279.613 | 1.397  (0.426-4.575) | 0.581 | 1.437  (0.436-4.738) | 0.552 |
| U072, RAO | PreCOVID | 1 | 0 | 0 | 0 | 0 | 0 | 0 | 1 | 0 | 2 | 80.081 | 1  (Reference) | - | 1  (Reference) | - |
|  | PostCOVID | 0 | 0 | 0 | 0 | 0 | 0 | 0 | 0 | 1 | 1 | 46.602 | 0.582  (0.053-6.417) | 0.658 | 0.578  (0.052-6.464) | 0.656 |
